# Cervical Intradural Extramedullary Endodermal Cyst Rapidly Manifesting Tetraparesis in a Pediatric Patient. A Case Report

**DOI:** 10.7759/cureus.79899

**Published:** 2025-03-01

**Authors:** Akira Teshigahara, Kenji Takagi, Hiroto Kawano, Yoshinori Maki, Kazumichi Yoshida

**Affiliations:** 1 Department of Neurosurgery, Shiga University School of Medicine, Otsu, JPN; 2 Neurosurgery, Hikone Chuo Hospital, Hikone, JPN; 3 Department of Neurosurgery, Shiga University of Medical Science, Shiga, JPN

**Keywords:** cervical, endodermal cyst, laminectomy, pediatric, posterior approach

## Abstract

Endodermal cyst (EC) is a rare benign congenital central nervous system tumor. As a common lesion of this entity, the cervical and cervicothoracic regions are reported. This cyst can be generally symptomatic in adult cases. Although symptomatic ECs can be found in pediatric cases, a male predominance was reported. Previously, the number of pediatric female cases with ECs in lower cervical levels seems limited. A four-year-old girl was referred to our hospital, complaining of neck pain, gait disturbance, and motor weakness in the bilateral upper and lower extremities. The symptoms worsened rapidly within two weeks. Magnetic resonance images revealed an intradural extramedullary cystic lesion at C4-C7 levels, which extremely compressed the spinal cord posteriorly. To improve the clinical symptoms of the patient, a right partial laminotomy via the posterior approach and partial removal of the cystic wall was performed. The histopathological diagnosis corresponded to EC. After the operation, the neurological deficits disappeared. Any recurrence of the EC was not observed two years after surgery. We described a female pediatric case of middle and lower cervical intradural extramedullary EC. In this case, rapidly progressive neurological deficits resulted from the EC compressing the spinal cord. In this report, we summarize the previous similar pediatric cases of middle and lower cervical ECs.

## Introduction

Endodermal cyst (EC) (also called “neurenteric cyst” or “enterogenic cyst”) is a relatively rare congenital entity that accounts for approximately 1.0 % of spinal tumors [1-3]. An EC can be generated when the anomalous connection between the endoderm and ectoderm persists during the third week of gestation [4, 5]. An EC can occasionally be associated with vertebral dysplasia [3]. Histopathologically, EC is featured with epithelium composed of mucin-producing simple columnar or cuboidal ciliated cells and non-ciliated goblet cells [1, 6].

An EC typically can become a symptomatic lesion in the second to fourth decades of life when EC grows and compresses the neural structures. An EC is accompanied by male dominance based on previous reports [7, 8]. This cyst in pediatric patients seems relatively rare compared to adult cases [7]. A common region of EC is an intradural extramedullary space ventrally to the spinal cord in the lower cervical and thoracic levels [6, 7, 9]. An EC can rarely result in aseptic meningitis, but typical manifestations related to EC occur, such as myelopathy or radiculopathy, because of the mass effect of the cyst and compression of the spinal cord [3]. Surgical treatment is the gold standard management of symptomatic ECs [6], although there is still a lack of a standardized approach (anterior or posterior approach).

Pediatric cases with symptomatic intradural extramedullary ECs in the middle and lower cervical regions have been described to date, but the rarity of EC in such a population and regions remains. Here, we report a pediatric case of a symptomatic intradural extramedullary EC in the middle and lower cervical regions surgically managed, referring to the previous literature.

## Case presentation

**Figure 1:**
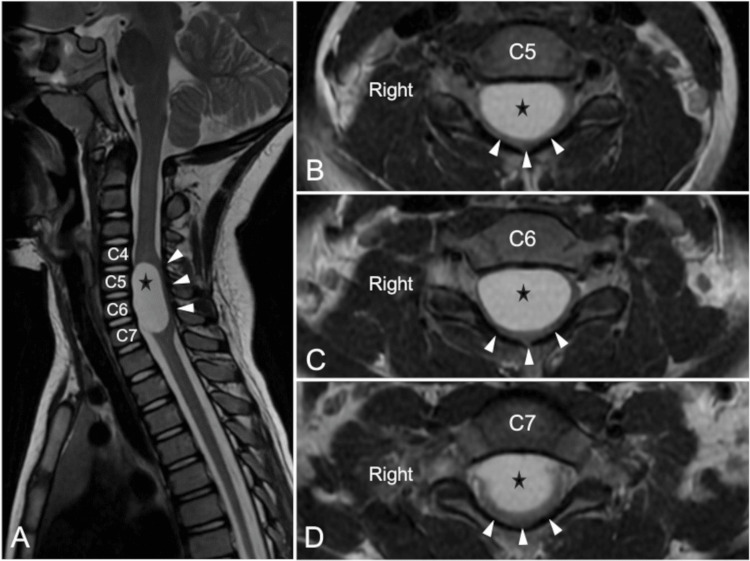
Preoperative cervical magnetic resonance images An intradural extramedullary cystic lesion (black star) at C4-C7 was disclosed. The lesion (black star) caused significant anterior displacement and compression of the spinal cord (white arrowheads), as evidenced by signal changes on T2-weighted imaging (A: a sagittal T2-weighted image; B-D: axial T2-weighted images).

A four-year-old girl was accompanied by her parents, complaining of neck pain, gait disturbance, and motor weakness in the bilateral upper and lower extremities. She had no previous medical or traumatic events. The symptoms were progressive for two weeks. Her physical examination revealed quadriparesis, corresponding to a manual muscle test (MMT) 3. The patient was hospitalized for further examinations. A blood sample examination did not reveal any inflammation. Because we suspected a lesion in the cervical spine, we performed a cervical magnetic resonance imaging examination. The exam revealed an intradural extramedullary cystic lesion (12.12 mm × 18.85 mm × 27.78 mm) from C4 to C7 levels. The lesion showed high signal intensity on T2-weighted magnetic resonance images (Figure 1). Flow voids, suggestive of a vascular lesion, were not observed.

The cyst seemed to be in the intradural and extramedullary space. The cervical spinal cord was extremely compressed by the cyst anteriorly located. Any other congenital anomalies, such as spina bifida, hemivertebrae, fused vertebral bodies, or Klippel-Feil anomaly, were not identified. As we thought the neurological symptoms of the patient could have resulted from the cyst, surgical management was planned.

**Figure 2:**
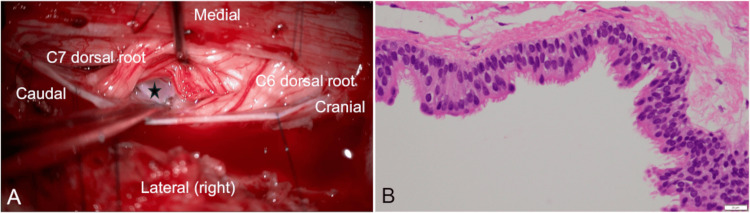
A representative intraoperative and histopathological images (A) An elastic and whitish cystic lesion (black star) was identified ventrally to the spinal cord; (B) Hematoxylin-eosin staining of the specimen (original magnification ×400). The cyst was composed of the mucus-producing, ciliated columnar epithelium. The epithelium contained no atypical cells.

Under general anesthesia, the patient was set in a prone position. The motor and somatosensory evoked potentials were prepared as intraoperative monitoring. We selected a posterior approach to minimize the operative invasiveness and avoid spinal instability and deformity after surgery. The right unilateral laminectomy was performed at C4 to C7 levels. The bulging dura mater was opened. Gently retracting the spinal cord, a cystic lesion was revealed ventrally. The cyst wall appeared firm, whitish, and elastic, without overt vascularization (Figure 2A). The rupture of the cyst released a cerebrospinal fluid-like leakage. The cyst wall was resected except for the dorsal region adhering to the spinal cord so as not to damage the spinal cord. The dura was sutured, and the wound was closed.

The postoperative course was uneventful. A few days after the operation, spontaneous movements of the right lower extremity began to return. Two weeks after surgery, the patient could walk independently and improved the impairment of the dexterity of the right hand. Histopathologically, the cervical cyst was composed of the mucus-producing, ciliated columnar epithelium. The epithelium contains no atypical cells. These findings were suggestive of the endodermal cyst (Figure 2B).

**Figure 3:**
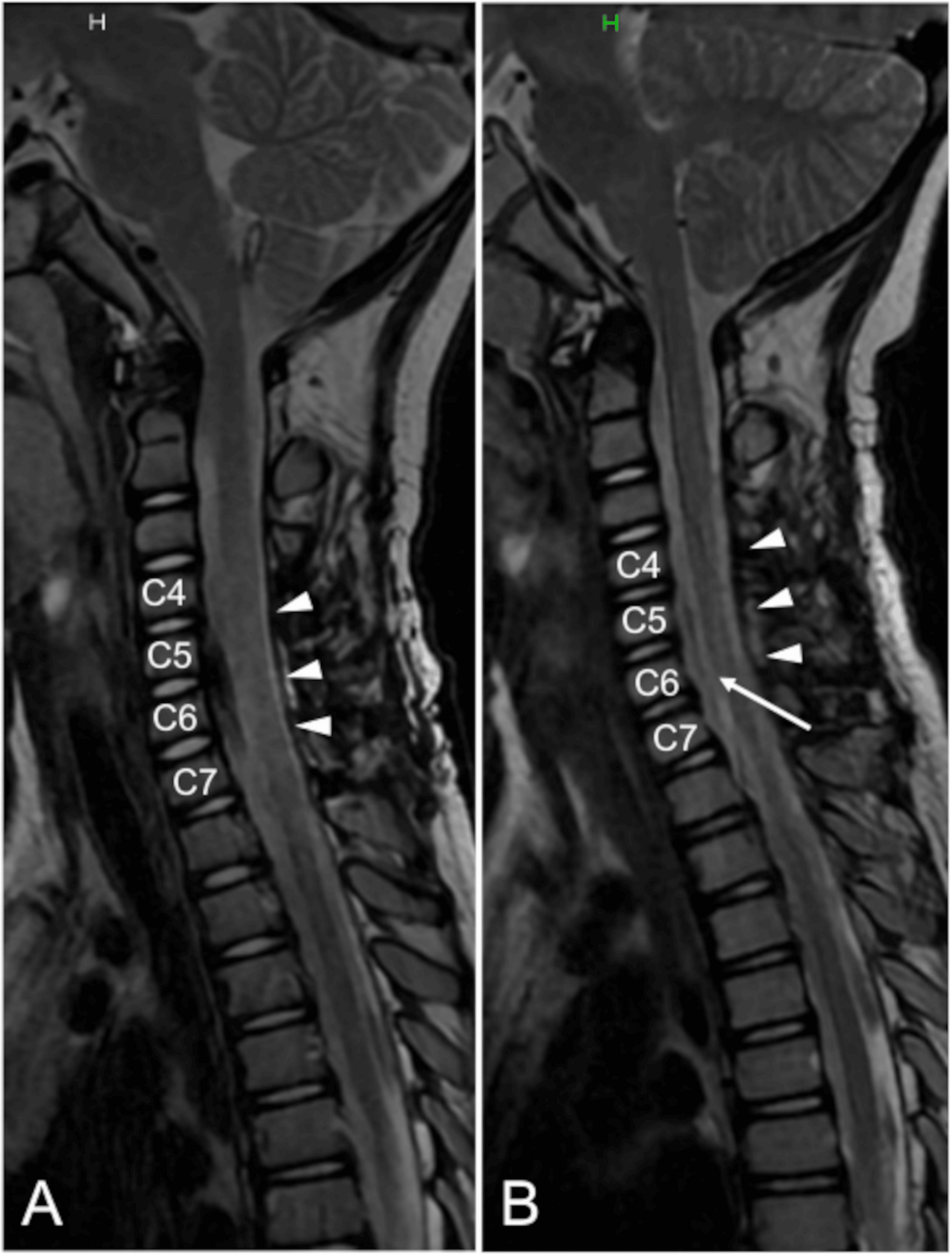
Postoperative cervical magnetic resonance images The compression of the spinal cord was resolved postoperatively (white arrowheads) (A). Any recurrence of the cyst was not observed two years after surgery (white arrow) (B) (sagittal T2-weighted images, A: one week after surgery and B: two years after operation).

The patient was discharged from the hospital three weeks after surgery. Preoperative symptoms disappeared one and a half months after surgery. A follow-up MRI examination two years after the operation showed no recurrence of the cyst (Figure 3).

The patient is still neurologically intact.

## Discussion

We described a pediatric case of an intradural extramedullary EC located at C4-C7 levels, which rapidly manifested motor weakness within two weeks. Unilateral laminectomy and removal of the EC via a posterior approach successfully resolved the compression of the spinal cord, and no recurrence was observed two years after surgery. Because the rapid neurological deficits appeared in this case, early diagnosis and timely surgical intervention were necessary to avoid neurological sequelae.

As for the diagnosis of EC, the following cystic lesions should be differentiated: arachnoid, dermoid/epidermoid, and neuroepithelial (colloid) cysts. Although the ventral location of an arachnoid cyst was described [10], this cyst is typically in the posterior arachnoid space to the spinal cord [11]. A neuroepithelial (colloid) cyst is frequently identified in the anterior half of the third ventricle, and the fourth and lateral ventricles follow this location. The middle and lower cervical regions seem very rare as a location of a neuroepithelial (colloid) cyst [12]. Dermoid/epidermoid cysts can be generated from dermal components of the first and second brachial arches. In the cervical regions, those cysts are typically located at the base of the tongue and beneath the skin within the subcutaneous tissues of the anterior neck [13]. In our case, the cyst was located in the middle and lower cervical regions, ventrally to the spinal cord. The cyst location was a common characteristic of ECs [6, 7, 9]. The pathological findings corresponded to the diagnosis of EC. Because preoperative magnetic resonance images did not show any flow voids, we did not think that the lesion could be a vascular anomaly. Therefore, we did not perform spinal angiography.

Focusing on pediatric cases of intradural extramedullary ECs in the middle and lower cervical regions since 2000, only several cases have been described (Table 1) [1, 2, 9, 14-16].

**Table 1:** Summary of endodermal cysts in middle and lower cervical levels in pediatric cases

	Age	Sex	Location level	Clinical symptoms	Duration	Treatment	Follow-up period	Recurrence	Additional treatment
Hicdonmez et al., 2004 [14]	6 years	Male	C4-6	Torticollis and sudden neck pain	3 weeks	Osteoplastic laminectomy (C3-C7), excision of the cyst wall	2 years	-	
Wakisaka et al., 2004 [15]	6 months	Female	C6-7	Cough and stridor	Two weeks	Posterior cervical approach (not detailed), right thoracotomy, cyst dissection	4 years	-	
Ito et al. 2011 [1]	14 years	Female	C6-7	Neck and shoulder pain, vesicorectal disturbance	Two months	C6 corpectomy, anterior spinal fusion (titanium plate and screws, and iliac bone)	18 months	-	Plate removal (1.5 years after surgery)
Al-Ahmed et al., 2013 [2]	4 years	Male	C5-6	Not detailed	Not detailed	C5 corpectomy, complete cyst removal, bone graft refixation (revision surgery, three months after the first surgery)	Not detailed	-	
Al-Ahmed et al., 2013 [2]	14 years	Male	Not detailed (described only cervical)	Not detailed	Not detailed	Laminectomy, total cyst removal		+	Ommaya reservoir insertion
Kozak et al., 2019 [16]	8 years	Male	C5-7	Severe quadriparesis	Within 24 hours	Laminectomy, subtotal resection	6 months	-	
Yamada et al., 2020 [9]	4 years	Female	C3-C5	Severe neck pain, urination difficulty	One week	Hemilaminectomy (C2-C6), cyst wall puncture (the first surgery), cyst wall partial resection (revision surgery, two months after the first)	11 years	+	Observation
Our case	4 years	Female	C4-C7	Neck pain, quadriparesis	Two weeks	Hemilaminectomy (C4-C7), cyst wall partial resection	2 years	-	None

Hicdonmez et al. reported a case of an intradural extramedullary EC at C4-C6 levels, manifesting torticollis and neck pain lasting for three weeks. In their case, the preceding hemorrhagic event in the EC aggravated neurological symptoms. C3-C7 laminectomy and cyst wall excision were effective, and the EC did not recur two years after surgery [14]. Wakisaka et al. reported a six-month-old girl manifesting cough and stridor for two weeks. An intradural extramedullary EC at C6-C7 levels was identified on magnetic resonance images. Their unique case was that a giant mediastinal cyst connecting to the duodenum accompanied the intradural extramedullary EC. The giant mediastinal cyst was also connected to the C7 spinal column with a fibrous cord [15]. Ito et al. also reported a 14-year-old girl with an intradural extramedullary EC at C6-C7 levels. They selected the anterior approach for the EC and performed a corpectomy of C6, removal of the EC, and anterior fusion using a harvested iliac bone, titanium plate, and screws. The plate was removed 1.5 years after surgery [1]. Al-Ahmed et al. described 11 pediatric cases of ECs, and two of those cases were ECs in the cervical regions. An anterior approach combined C5 corpectomy and complete cyst excision was selected for a 4-year-old boy, but revision surgery for bone graft dislodgement was necessary. Although Al-Ahmed et al. mentioned the effectiveness of the anterior approach for ECs, they also alerted to the following risks, such as damage to adjacent neurovascular structures, fusion failure, hematoma formation, and cerebrospinal fluid leakage [2]. A posterior approach, including laminectomy and total cyst excision, was initially performed, and an Ommaya reservoir was additionally inserted for a recurrence of an EC [2]. Kozak et al. reported a case of an eight-year-old boy whose severe quadriparesis suddenly worsened due to an intradural extramedullary EC at C5-C7 [16]. The EC in their case was managed with laminectomy and cyst wall resection. They left intentionally the ventral part of an EC strongly adhered to the spinal cord. No recurrence was observed six months after surgery [16]. A four-year-old girl with an intradural extramedullary EC at C3-C5 levels was initially managed with hemilaminectomy at C2 to C6 and cyst wall puncture. However, a recurrence of the EC appeared two months later, and partial removal of the cyst wall except for the string adhesion to the spinal pia mater was additionally performed. Although another recurrence was observed on follow-up magnetic resonance images, spontaneous shrinkage of the cyst resulted in an unnecessary additional operation [9].

The intradural extramedullary EC in our case was successfully managed with hemilaminectomy and partial removal of the cyst wall. Overaggressive removal of ECs is not recommended to avoid postoperative complications [9, 16]. Considering the benign nature of ECs, subtotal resection of ECs, not complete resection, can achieve satisfactory outcomes [9, 16]. The anterior approach can be beneficial for complete cyst removal; however, this approach requires vertebral corpectomy and fusion [1, 2]. Considering the invasiveness of the anterior approach in the long-term period during the patient's development, it seems controversial to select this surgical fashion for all pediatric cases. Revision surgery, or secondary surgery to remove inserted instrumentation, can also be a stress for patients [1,2]. The posterior approach (laminectomy or hemilaminectomy) can be another option for ECs [2, 9, 14, 15]. This operation limits complete cystic removal because the spinal cord exists dorsally to ECs. In addition, muscle detachment and bilateral laminectomy may result in secondary cervical deformity. In our case, we selected hemilaminectomy to minimize the muscle sacrifice and avoid the invasiveness related to the anterior approach. Cystic drainage such as an Ommaya reservoir [2] or a shunt tube may also be a good choice, but we did not select the surgery to avoid an additional surgery resulting from the obliteration of an inserted device.

As pediatric patients have long lifetimes, avoiding postoperative complications seems essential in treating benign lesions such as ECs. A recurrence of an intradural extramedullary EC appeared within a short period, as in the case of Yamada et al.. Thus, only the cyst wall puncture does not seem enough to manage ECs [9]. Because partial resection of ECs may result in a high recurrence ratio of 37% during a 30-year follow-up [6], patients should be followed for as long as possible. Even when a recurrence of ECs is observed during follow-up periods, spontaneous shrinkage of lesions might occur [9]. Therefore, the necessity of an additional operation should be prudently evaluated in each patient.

## Conclusions

We reported a pediatric case of an intradural extramedullary EC in the middle and lower cervical regions, which was successfully treated with a posterior cervical approach. No recurrence was observed at the follow-up two years after surgery, which might have resulted from the limited follow-up period. Therefore, the patient should be regularly monitored for a long-term period. As we consider the lifetime expectancy of pediatric patients after surgery, an operative strategy should be planned to avoid a recurrence of EC and postoperative complications. In our case, hemilaminectomy and partial removal of the cyst wall resulted in the resolution of preoperative neurological symptoms. As a recurrence of EC more than 10 years after surgery was reported in a previous study, long-term follow-up based on neurological examinations should be warranted after surgery. If a recurrence of EC is suspected, a magnetic resonance imaging examination should be considered. Even when a recurrence of EC is radiologically observed, additional surgery should be indicated based on neurological symptoms. Only observation might be enough when EC shrinks spontaneously following recurrence.
